# A Model for Estimating Biological Age From Physiological Biomarkers of Healthy Aging: Cross-sectional Study

**DOI:** 10.2196/35696

**Published:** 2022-05-10

**Authors:** Karina Louise Skov Husted, Andreas Brink-Kjær, Mathilde Fogelstrøm, Pernille Hulst, Akita Bleibach, Kaj-Åge Henneberg, Helge Bjarup Dissing Sørensen, Flemming Dela, Jens Christian Brings Jacobsen, Jørn Wulff Helge

**Affiliations:** 1 Xlab, Center for Healthy Aging Department of Biomedical Sciences University of Copenhagen Copenhagen Denmark; 2 Department of Physiotherapy and Occupational Therapy University College Copenhagen Copenhagen Denmark; 3 Digital Health Department of Health Technology Technical University of Denmark Lyngby Denmark; 4 Biomedical Engineering Department of Health Technology Technical University of Denmark Lyngby Denmark; 5 Department of Geriatrics Bispebjerg and Frederiksberg Hospital Copenhagen Denmark; 6 Department of Biomedical Sciences Faculty of Health and Medical Sciences University of Copenhagen Copenhagen Denmark

**Keywords:** biological age, model development, principal component analysis, healthy aging, biomarkers, aging

## Abstract

**Background:**

Individual differences in the rate of aging and susceptibility to disease are not accounted for by chronological age alone. These individual differences are better explained by biological age, which may be estimated by biomarker prediction models. In the light of the aging demographics of the global population and the increase in lifestyle-related morbidities, it is interesting to invent a new biological age model to be used for health promotion.

**Objective:**

This study aims to develop a model that estimates biological age based on physiological biomarkers of healthy aging.

**Methods:**

Carefully selected physiological variables from a healthy study population of 100 women and men were used as biomarkers to establish an estimate of biological age. Principal component analysis was applied to the biomarkers and the first principal component was used to define the algorithm estimating biological age.

**Results:**

The first principal component accounted for 31% in women and 25% in men of the total variance in the biological age model combining mean arterial pressure, glycated hemoglobin, waist circumference, forced expiratory volume in 1 second, maximal oxygen consumption, adiponectin, high-density lipoprotein, total cholesterol, and soluble urokinase-type plasminogen activator receptor. The correlation between the corrected biological age and chronological age was *r*=0.86 (*P*<.001) and *r*=0.81 (*P*<.001) for women and men, respectively, and the agreement was high and unbiased. No difference was found between mean chronological age and mean biological age, and the slope of the regression line was near 1 for both sexes.

**Conclusions:**

Estimating biological age from these 9 biomarkers of aging can be used to assess general health compared with the healthy aging trajectory. This may be useful to evaluate health interventions and as an aid to enhance awareness of individual health risks and behavior when deviating from this trajectory.

**Trial Registration:**

ClinicalTrials.gov NCT03680768; https://clinicaltrials.gov/ct2/show/NCT03680768

**International Registered Report Identifier (IRRID):**

RR2-10.2196/19209

## Introduction

Biological age (BA) is a measure that quantifies where an individual is on the aging trajectory, assessed by the physiological profile, in comparison with the average person of that given chronological age (CA) within the population from which the equation was generated [1,2]. The predictive abilities of BA have been investigated in relation to age-related diseases such as cardiovascular disease (CVD) and type 2 diabetes (T2D) and some BA models have been found to predict mortality better than CA [3-5]. Parallels can be drawn between the changes that occur with aging and the changes that occur with an unhealthy lifestyle (especially related to physical inactivity and obesity) and the risk of developing CVD and T2D [6,7]. Therefore, the objective assessment of BA is an appealing approach for risk stratification and health literacy within public health promotion. However, truly measuring the current state of aging, and thereby objectively determining BA, would entail studies that follow people until they die and biomarkers representing all bodily functions. This is practically impossible and objectively unfeasible for use in a clinical setting. To circumvent this, BA models conceptualizing some mechanisms of aging are proposed as surrogate measures of BA. Despite a substantial research effort [8-10], there is still no agreement upon which panel of biomarkers to use when defining BA [11]. Targeting health promotion and management of lifestyle-related diseases, studies have developed several BA models that evaluate the degree of severity of the metabolic syndrome [12], the relation to waist circumference [13], the relation to physical fitness level [14,15], and the organ-specific health status [16], just to mention a few.

Increasing life expectancy and low fertility rates will have a profound impact on future resources and health care needs [17,18]. Forecasts anticipate that by 2050, people aged 65 years or above will constitute more than 20% of the population worldwide [19,20]. This is the decade in life where chronic diseases (eg, CVD, cancer, and T2D) frequently manifest [21], making healthy aging a key objective for research [22-24]. Healthy aging is defined as an extension of health span [25] also characterized by the “healthy aging phenotype” avoiding major chronic diseases as well as cognitive and physical impairments [22]. The important work from Lara and colleagues [26] has resulted in a panel of biomarkers of healthy aging. The purpose of our study was to apply a novel approach in order to incorporate biomarkers of healthy aging into a BA model. For this purpose, we used the first principal component (1PC) obtained from principal component analysis (PCA) as the method to assess individual BA. The goal was to create a BA model based on the healthy aging phenotype. In this way, the model can be used to identify those deviating from the healthy aging trajectory. Thus, no difference between average CA and estimated BA was expected in the study population of healthy individuals.

## Methods

### Participants

**Figure 1:**
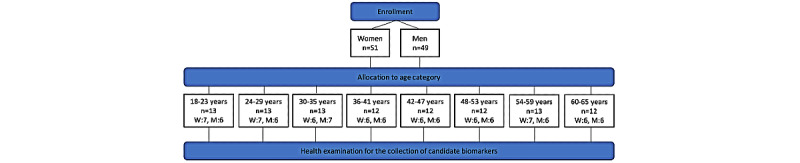
Flow chart of the allocation of enrolled participants in age categories. W: women; M: men.

We included 100 healthy Danish individuals, 51 women and 49 men, between 18 and 65 years of age, to participate in an extensive health examination and the data collection of candidate biomarkers for the BA model. We recruited an equal number of women and men in each 5-year age category (Figure 1).

### Ethics Approval

The study was approved by the Regional Ethics Committee, Denmark (H-18031350), recorded as a Clinical Trial (Clinical Trial number: NCT03680768), and performed in accordance with the Helsinki declaration. Participants were informed orally and in writing about the study protocol and the potential risks before obtaining written consent.

### Candidate Biomarkers

On the day of the health examination, participants came to the laboratory following an overnight fast and having avoided exercise activities and alcohol consumption for 24 hours and restrained from smoking for at least 4 hours. Information on the participants’ previous and current health status included weekly alcohol consumption, smoking habits, present medications, past medical history, and self-administered questionnaires on physical activity level (Physical Activity Scale 2.1) [27] and quality of life (12-item Short Form version 2 [SF-12v2]). We gathered data on the candidate biomarkers listed in Table 1. These 32 variables are all physiological components of healthy aging that are associated with aging, age-related diseases, and are affected by changes in lifestyle. In addition, this panel of biomarkers covers multiple areas of human function, and they are suitable to study in humans in vivo. For a more comprehensive description of the rationale for including these 32 variables as candidate biomarkers, we refer to our protocol paper (Clinical Trial number: NCT03680768) [28].

**Table 1:** Candidate biomarkers measured in the study participants (n=100) showing means with SDs and outcome units per year increase (regression slope with 95% CI).

Biomarkers^a^			Mean (SD)		Slope (CI)
**Body composition**					
	Weight (kg)	75.7 (13.1)		0.03 (–0.2 to 0.2)	
	Waist circumference (cm)	83.4 (9.8)		0.2 (0.05 to 0.3)	
	Hip circumference (cm)	101.4 (7.1)		–0.001 (–0.1 to 0.1)	
	Waist/hip ratio	0.8 (0.07)		0.002 (0.001 to 0.003)	
	Fat mass (%)	26.8 (8.3)		0.09 (–0.03 to 0.2)	
	Muscle mass (kg)	52.8 (10.9)		–0.05 (–0.2 to 0.1)	
**Metabolic health**					
	Fasting blood glucose (mmol/l)	5.1 (0.4)		0.01 (0.004 to 0.015)	
	HbA_1c_^b^ (mmol/mol)	32.8 (3.2)		0.12 (0.08 to 0.16)	
	AGEs^c^ (AU)	1.8 (0.5)		0.027 (0.022 to 0.031)	
	Insulin (pmol/l)	44.4 (25.3)		0.05 (–0.32 to 0.42)	
	Triglycerides (mmol/l)	0.9 (0.4)		0.002 (–0.004 to 0.008)	
	Free fatty acids (μmol/l)	440 (212)		2.36 (–0.72 to 5.46)	
	Leptin (pg/ml)	8411 (9472)		–60.0 (–199.8 to 79.9)	
	Adiponectin (mg/ml)	11515 (6490)		106.6 (13.4 to 199.8)	
	HDL^d^ (mmol/l)	1.5 (0.4)		0.01 (0.006 to 0.017)	
	LDL^e^ (mmol/l)	2.8 (0.8)		0.02 (0.01 to 0.03)	
	TC^f^ (mmol/l)	4.5 (0.9)		0.03 (0.02 to 0.04)	
	TC/HDL ratio	3.1 (0.9)		0.003 (–0.01 to 0.02)	
**Immune function**					
	CRP^g^ (mg/l)	1.6 (3.4)		–0.04 (–0.09 to 0.01)	
	suPAR^h^ (ng/ml)	2.09 (0.5)		0.01 (0.003 to 0.017)	
**Cell blood count**					
	Hemoglobin (mmol/l)	8.7 (0.8)		0.004 (–0.01 to 0.02)	
	Hematocrit (%)	41.6 (3.8)		0.03 (–0.03 to 0.09)	
**Cardiorespiratory function**					
	Diastolic BP^i^ (mmHg)	78.0 (10.1)		0.4 (0.3 to 0.5)	
	Systolic BP (mmHg)	124.2 (16.7)		0.6 (0.3 to 0.8)	
	FEV_1_^j^ (L)	3.9 (0.9)		–0.02 (–0.04 to –0.01)	
	FVC^k^ (L)	4.9 (1.0)		–0.02 (–0.04 to –0.01)	
	FEV_1_/FVC ratio (%)	77.8 (11.6)		–0.13 (–0.20 to –0.05)	
**Physical capacity**					
	VO_2max_^l^ (ml/minute/kg)	39.3 (8.11)		–0.18 (–0.28 to –0.06)	
	STS^m^ (stands)	23.4 (5.2)		–0.07 (–0.14 to 0.01)	
	Handgrip strength (kg)	36.0 (9.4)		–0.8 (–0.2 to 0.1)	
	Biceps strength (kg)	35.0 (11.5)		–0.1 (–0.3 to 0.03)	
	Quadriceps strength (Nm)	152.4 (51.3)		–0.7 (–1.4 to 0.1)	

^a^Missing values were present in leptin (n=99), CRP (n=87), hematocrit (n=97), hemoglobin (n=99) and bicep’s strength (n=98).

^b^HbA_1c_: glycated hemoglobin type A_1c_.

^c^AGE: advanced glycation end product.

^d^HDL: high-density lipoprotein.

^e^LDL: low-density lipoprotein.

^f^TC: total cholesterol.

^g^CRP: C-reactive protein.

^h^suPAR: soluble urokinase plasminogen activator receptor.

^i^BP: blood pressure.

^j^FEV_1_: forced expiratory volume in 1 second.

^k^FVC: forced vital capacity.

^l^VO_2max_: maximal oxygen consumption.

^m^STS: 30-second sit-to-stand chair rise.

### Procedures

Variables of *body composition* were measured by dual-energy X-ray absorptiometry scanning (Lunar Prodigy Advance; Lunar). Waist and hip circumference were measured twice using a standard measuring tape. Variables of *metabolic health* and *immune function* were measured from venous blood samples. We extracted plasma and stored it at –80°C before analysis. Plasma concentrations of C-reactive protein, total cholesterol (TC), low-density lipoprotein (LDL), high-density lipoprotein (HDL), triglycerides, free fatty acids, and glycerol were measured separately by spectrophotometry (Cobas 6000 c501; Roche). Plasma fasting blood glucose (FBG) concentration was measured on an automated analyzer (Hitachi 912; Roche). Plasma insulin, adiponectin, and leptin concentrations were measured by RIA kits (HADP-61HK; Millipore). Plasma concentrations of soluble urokinase plasminogen activator receptor (suPAR) were measured using the commercially available suPARnostic ELISA kit, according to the manufacturer’s instructions (ViroGates). Advanced glycation end products (AGEs) were measured noninvasively using an AGE reader (Diagnoptics Technologies). We measured glycated hemoglobin type A_1c_ (HbA_1c_) on whole blood using DCA Vantage Analyser (Siemens Healthcare) for the analysis. Resting arterial blood pressure (BP) was measured in triplicate (with 1-minute intervals) using an automatic monitor (Boso-medicus control). Forced vital capacity (FVC) and forced expiratory volume in 1 second (FEV_1_) were assessed by spirometer measurements (Vyntus SPIRO spirometer) with participants sitting on a chair and wearing a nose clip and mouthpiece. Initially, participants breathed normally before conducting a rapid maximal inspiration immediately followed by an expiration with a maximal effort that continued until no more air could be expelled while maintaining an upright posture. The procedure was repeated a minimum of 3 times and a maximum of 7. The trial with the highest reading was used and the Vyntus SPIRO software (SentrySuite) automatically assessed the repeatability, acceptability, and usability criteria defined by the American Thoracic Society and the European Respiratory Society [29]. The handgrip, biceps, and quadriceps strength were measured by a handheld dynamometer (Takei, A5401; Physical Company), a digital back strength dynamometer (Takei TKK 5402; Takei Scientific Instruments Co. Ltd.), and a handheld dynamometer (microFET2; Hoggan Health Industries, Inc.), respectively. At least three attempts were made until no rise in strength occurred. Each test was interspersed with 1-minute rest. Maximal oxygen consumption (VO_2max_) was measured by a graded exercise test, performed on a bicycle ergometer (Lode Corival) using breath-by-breath (Quark PFT Ergo; Cosmed) oxygen consumption measurements. After 5 minutes of warm-up at 50 and 100 W for women and men, respectively, the load increased by 25 W every minute until voluntary exhaustion. VO_2max_ was determined as the highest 30-second rolling average of VO_2_.

### Exclusion and Inclusion of Candidate Biomarkers

To observe the trajectory of normal healthy aging, we excluded participants diagnosed with or having a previous history of T2D, CVD, cancer, and thyroid dysfunction and who were free of the use of medication to lower cholesterol levels, glucose concentration, and BP [16,30-32]. In addition, a 99% reference interval (mean ±2.96×SD) was applied to examine any potential outliers [30]. To acknowledge age-related decrements within the healthy aging spectrum, however, extreme values below or above the reference interval were individually assessed [33]. We excluded the candidate biomarker AGE from the study due to technical problems affecting the reliability of the measurements.

The actual selection between the remaining 31 candidate biomarkers followed a systematic stepwise method in alignment with previous studies [3,30,34]. To begin with, all candidate biomarkers were submitted to Pearson correlation analysis to assess the strength and direction of association between CA and the candidate biomarkers. All biomarkers that were significantly correlated with CA (|*r*|>0.15; *P*≤.05) were included. To minimize redundancy arising in the analysis, we assessed intercorrelation between the included biomarkers. If the correlation between biomarkers was high (|*r*|≥0.7) and they have a similar clinical function, they are likely to be dependent on the same biological factor and one is excluded depending on the strength of the relationship with CA and the clinical relevance.

### Principal Component Analysis

PCA is a factor analysis that reduces dimensions but preserves most of the information in the original data set. PCA is a linear transformation that applies orthogonal rotation to find factors/principal components that capture the largest amount of information in the data [35]. As the PCA produces uncorrelated principal components disclosing which variables are most valuable for clustering the data, it can be used to elucidate the minimum numbers of candidate biomarkers necessary for estimating BA [36]. Traditionally, all principal components with an eigenvalue above 1 are included, or alternatively the number of principal components that together contain 80% of the variation in the data set. However, we will follow the approach first applied by Nakamura et al in 1988 [37] and applied by others since [12,30,31,38], and use the 1PC from the PCA to estimate individual BA.

To do so, included biomarkers were normalized to a mean of 0 and unit SD, which gives them equal weight in the PCA. The subsequent estimation of BA was performed in 3 steps. First, based on the PCA loading scores, a standardized individual BA score (BAS) was modeled:

BAS = w_0_ + (w_1_x_1_) + (w_2_x_2_) +^...^+ (w_N_x_N_) (1)

where *x* represents the original value of each of the *N* biomarkers (without units). The coefficient *w_n_* is defined as

w_n_ = loading score_n_/σ_n_ (2)

and the constant *w*_0_ as



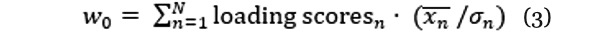



where *w_n_* represents each of the *N* biomarkers and 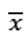 and *σ* represent the original mean and SD for each biomarker, respectively. The loading scores represent the contribution of each biomarker to 1 unit vector of the principal component.

Second, we transform the BA score into BA in units of years by application of the T-scale method [37]:



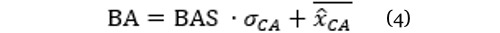



where *σ*_CA_ and 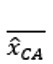 are the SD and mean of CA, respectively, of the sample size. However, this introduces a regression toward the mean effect (overestimation of younger individuals’ BA and underestimation of older individuals’ BA) [39], which is why the correction model proposed by Dubina et al [40] is applied:



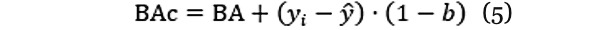



where BAc is the corrected biological age, *y_i_* represents individual CA, 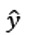 is the mean CA of the study sample, and represents the slope in the linear regression assessing the relationship between BA and CA.

### Statistics

We present candidate biomarkers as means with SDs and by linear regression to describe the direction and change of the candidate biomarkers per year. We assessed normal distribution using q-q plots and histograms, and checked variance of homogeneity and assessment of linearity by plotting residuals versus predicted values. Paired *t* test was used to assess differences within sex and the difference between BAc and CA (age difference) was calculated as CA – BAc. The statistical analyses were performed in SAS Enterprise Guide 7.1 and MATLAB R2018b. Statistical significance was considered at *P*≤.05 in all statistical tests.

## Results

### Systematic Stepwise Selection of Biomarkers

#### Correlation Analysis

**Figure 2:**
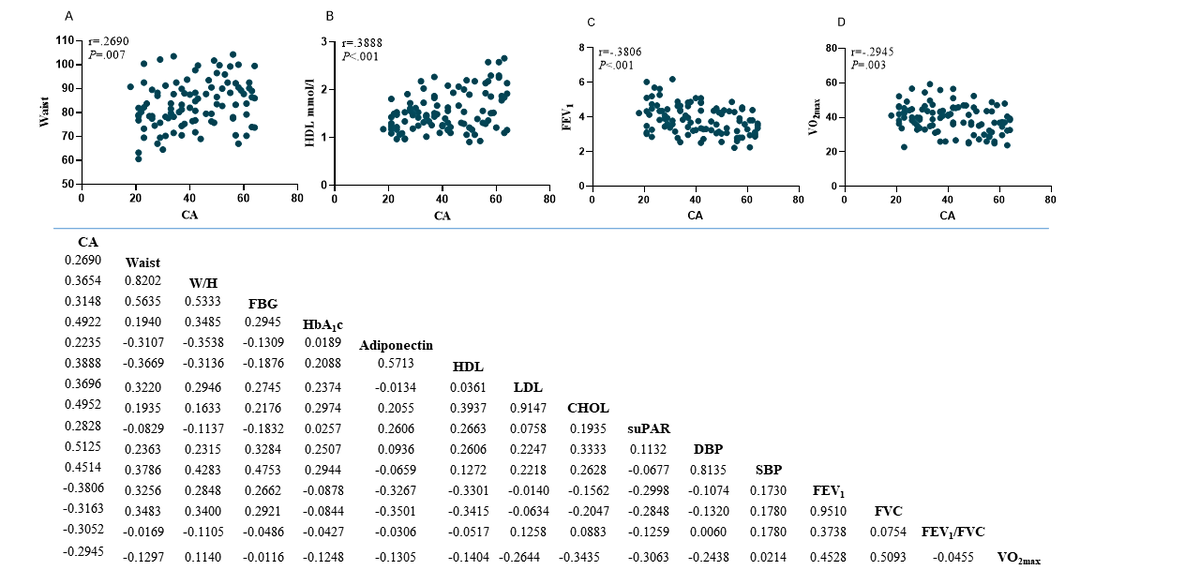
Top: Scatterplots and Pearson’s correlations of: waist circumference (A), high density lipoprotein (B), forced expiratory volume in 1. sec (C), maximal oxygen uptake (D). Bottom: Pearson’s correlation coefficients of the 15 biomarkers significantly correlated with age and their inter-correlations. CA: chronological age; W/H: waist to hip ratio; FBG: fasting blood glucose; HbA_1c_: glycated hemoglobin type A_1c_; HDL: High density lipoprotein; LDL: Low density lipoprotein; CHOL: total cholesterol; suPAR: soluble urokinase plasminogen activator receptor; DBP: Diastolic blood pressure; SBP: Systolic blood pressure; FEV_1_: Forced expiratory volume in 1. sec; VO_2max_: maximal oxygen uptake.

Pearson correlation coefficient was calculated for each of the 31 candidate biomarkers as a function of CA (Multimedia Appendix 1). Overall, 15 biomarkers substantially correlated with CA covering 5 domains. *Body composition* (waist circumference and waist/hip ratio), *metabolic health* (FBG, HbA_1c_, adiponectin, HDL, LDL, and TC), *immune function* (suPAR), *cardiorespiratory function* (diastolic and systolic BP, FEV_1_, FVC, FEV_1_/FVC ratio), and *physical capacity* (VO_2max_). We observed positive correlations in waist circumference, waist/hip ratio, FBG, adiponectin, HbA_1c_, HDL, LDL, TC, suPAR, diastolic BP (DBP), and systolic BP (SBP) and negative correlations for FEV_1_, FVC, FEV_1_/FVC ratio, and VO_2max_ (Figure 2).

#### Assessment of Redundancy

We observed high intercorrelations for some of the variables (Figure 2, bottom) and selected those with the strongest correlation with age or with the highest clinical significance within each cluster. Therefore, as FEV_1_, FVC, and FEV_1_/FVC ratio all represent pulmonary function and FEV_1_ has the highest correlation with age (*r*=–0.3806; *P*<.001) compared with FVC (*r*=–0.3163; *P*=.001) and FEV_1_/FVC (*r*=–0.3052; *P*=.002), FEV_1_ was selected. In the same manner we selected TC (*r*=0.4952; *P*<.001) over LDL (*r*=0.3696; *P*<.001). HbA_1c_ and FBG concentration are both markers of glycemic control, and a high correlation between HbA_1_c and FBG has been shown in people with and without T2D [41,42]. We suggest that the moderate intercorrelation (*r*=0.2945; *P*=.003) found in this study is due to the sample size. HbA_1c_, which shows a higher correlation with age, has previously been used in the literature in BA models [31] and is generally preferred over FBG due to its higher applicability in a clinical setting. Thus, to reduce redundancy, we only include HbA_1c_ as a marker of glycemic control despite an intercorrelation less than 0.7.

We observed a high intercorrelation between waist circumference and waist/hip ratio, the latter having the highest correlation with CA. Despite this, waist circumference was selected due to its strong association with visceral adipose tissue [43], its clinical importance as the best single anthropometric measure able to identify individuals at high risk of CVD and T2D, and its simplicity [44-46]. In addition, the inherent problem of the equation that an individual who is morbidly obese could have the same waist/height ratio as a normal-weight individual made us select waist circumference. Finally, DBP and SBP had an intercorrelation of *r*=0.8135 (*P*<.001), and a very similar correlation with age (*r*=0.5125; *P*<.001 and *r*=0.4514; *P*<.001, respectively). Instead, we calculated mean arterial pressure (MAP = 1/3SBP + 2/3DBP) to capture both parameters. MAP had a correlation with age of *r*=0.510 (*P*<.001) and an intercorrelation with SBP and DBP of *r*=0.943 (*P*<.001) and *r*=0.961 (*P*<.001), respectively. Thus, a total of 9 biomarkers were submitted to the PCA: waist circumference, FEV_1_, HbA_1c_, adiponectin, HDL, TC, suPAR, MAP, and VO_2max_ (scatterplots and Pearson correlation with age for all 9 biomarkers are available in Multimedia Appendix 2).

### Applying PCA

Following the normalization of the data set comprising the 9 biomarkers, we applied PCA for women and men separately, with and without the inclusion of CA. By including and excluding CA, we could assess if the direction of the 1PC was similar in both cases, thus assuming that the 1PC can be seen as a general aging factor. The analysis showed high loading scores for CA on the 1PC for both women and men (0.473 and 0.515, respectively), confirming the close relationship between age and 1PC (Table 2). In the second PCA, we excluded CA and found that the relationship between the 9 biomarkers and the 1PC persisted. The 1PC had eigenvalues above 1.0 and accounts for 30.96% (females) and 25.04% (males) of the total variance in the battery of 9 biomarkers (Table 3). These results indicate that the 9 biomarkers reflect underlying measures of a healthy aging trajectory.

To clarify how the variables contribute to the estimation of the BA model, we calculated the percentage contribution of each variable using the following equation:



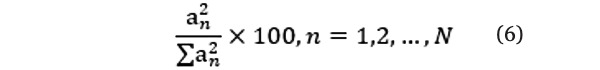



where a^2^_n_ is the given loading score and *N* is the number of variables (Table 3). In women, TC concentration contributed the most (21.8%) followed by MAP (18.9%) and HbA_1c_ (16.7%). For men, waist circumference contributed the most (24.1%) closely followed by VO_2max_ (22.6%) and TC concentration (14.5%).

**Table 2:** The linear combination of normalized variables for the 1PC by gender (chronological age included).

Principal component analysis variables	Loading scores for 1PC^a^		
	Women		Men
Chronological age	0.473		0.515
Mean arterial blood pressure^b^	0.392		0.294
Glycated hemoglobin	0.348		0.352
Waist circumference	0.144		0.378
Forced expiratory volume in 1 second	–0.164		–0.340
Maximal oxygen consumption	–0.287		–0.321
Adiponectin	0.199		0.078
High-density lipoprotein	0.346		0.127
Total cholesterol	0.405		0.337
suPAR^c^	0.220		0.167
Eigenvalue^d^	3.50	2.90	
Explained variance %^e^	35.04	28.96	

^a^1PC: first principal component comprising the best fit line with the largest sum of squares distances.

^b^Mean arterial blood pressure = (1/3SBP + 2/3DBP), where SBP is systolic blood pressure and DBP is diastolic blood pressure.

^c^suPAR: soluble urokinase plasminogen activator receptor.

^d^Eigenvalue: the sum of squared distances for 1PC.

^e^Explained variance %: how many percent does the 1PC explain of the total variance in the data set.

**Table 3:** The linear combination of normalized variables for the 1PC^a^ by gender (chronological age excluded) and the relative contribution of each physiological variable to BA^b^ estimation.

	Women			Men	
	Loading scores	Contribution (%)	Loading scores		Contribution (%)
Mean arterial blood pressure^c^	0.435	18.9	0.349		12.2
Glycated hemoglobin	0.408	16.7	0.324		10.5
Waist circumference	0.173	3.0	0.491		24.1
Forced expiratory volume in 1 second	–0.138	1.9	–0.309		9.5
Maximal oxygen consumption	–0.341	11.6	–0.475		22.6
Adiponectin	0.228	5.2	–0.046		0.2
High-density lipoprotein	0.390	15.2	–0.020		0.04
Total cholesterol	0.467	21.8	0.3804		14.5
suPAR^d^	0.238	5.7	0.254		6.4
Eigenvalue^e^	2.79	N/A^f^	2.25		N/A
Explained variance %^g^	30.96	N/A	25.04		N/A

^a^1PC: first principal component comprising the best fit line with the largest sum of squares distances.

^b^BA: biological age.

^c^Mean arterial blood pressure = (1/3SBP + 2/3DBP), where SBP is systolic blood pressure and DBP is diastolic blood pressure.

^d^suPAR: soluble urokinase plasminogen activator receptor.

^e^Eigenvalue: the sum of squared distances for 1PC.

^f^N/A: Not applicable.

^g^Explained variance %: how many percent does the 1PC explain of the total variance in the data set.

### Biological Age Model

By applying Equation 1, the loading scores from the PCA were used to construct individual standardized BAS as a function of the 9 biomarkers as shown in the following equations:

BAS_female_ = –11.04 + (0.03MAP) + (0.126HbA_1c_) + (0.018Waist) – (0.018FEV_1_) – (0.053VO_2max_) + (3.205·10^–5^·Adiponectin) + (0.909HDL) + (0.500TC) + (0.400suPAR)​​

BAS_male_ = –11.23 + (0.037MAP) + (0.103HbA_1c_) + (0.066Waist) – (0.431FEV_1_) – (0.067VO_2max_) – (1.058·10^–5^·Adiponectin) – (0.062HDL) + (0.442TC) + (0.828suPAR)

Subsequently, the BAS was scaled by applying Equation 4.

BA_female_ = (BAS × 13.6) + 41.3

BA_male_ = (BAS × 13.8) + 41.1

Scaling the score into units of years makes it more feasible to use when applying it to health promotion in the general population. Introducing this relationship between CA and BA has been shown to create some bias at the regression ends. Thus, following the previously mentioned correction model of Dubina et al [40] (Equation 5), the final BA models are expressed as

BAc_female_ = –56.67 + (0.27MAP) + (1.02HbA_1c_) + (0.1453Waist) – (2.03FEV_1_) – (0.43VO_2max_) + (0.0003·Adiponectin) + (7.39HDL) + (4.06TC) + (3.24suPAR) + (0.20CA)

BAc_male_ = –70.37 + (0.34MAP) + (0.95HbA_1c_) + (0.60Waist) – (3.96FEV_1_) – (0.62VO_2max_) – (9.73·10^–5^·Adiponectin) – (0.57HDL) + (4.06TC) + (7.61suPAR) + (0.32CA)

**Figure 3:**
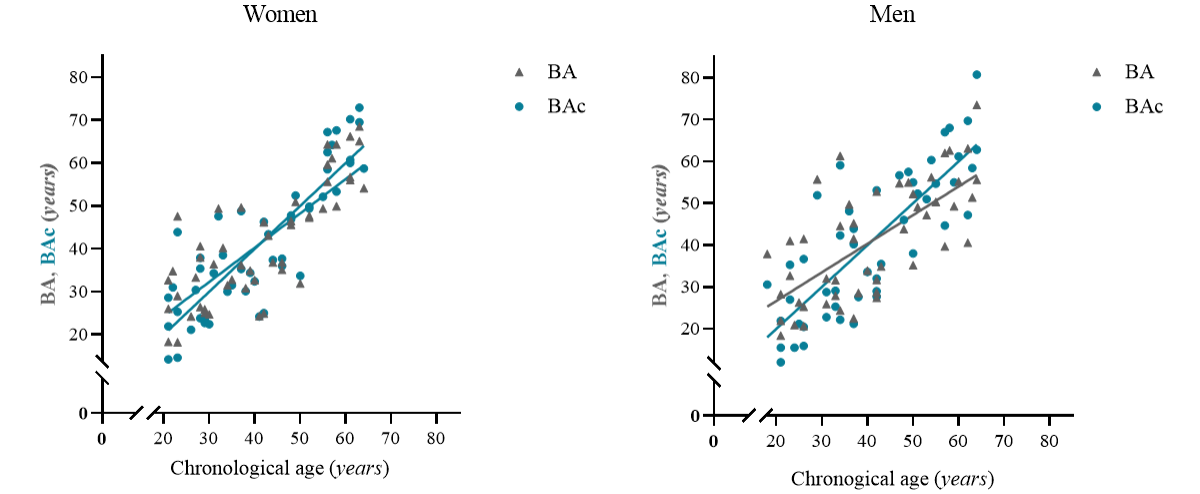
Regression lines before (BA) and after (BAc) correction for women and men, respectively.

**Figure 4:**
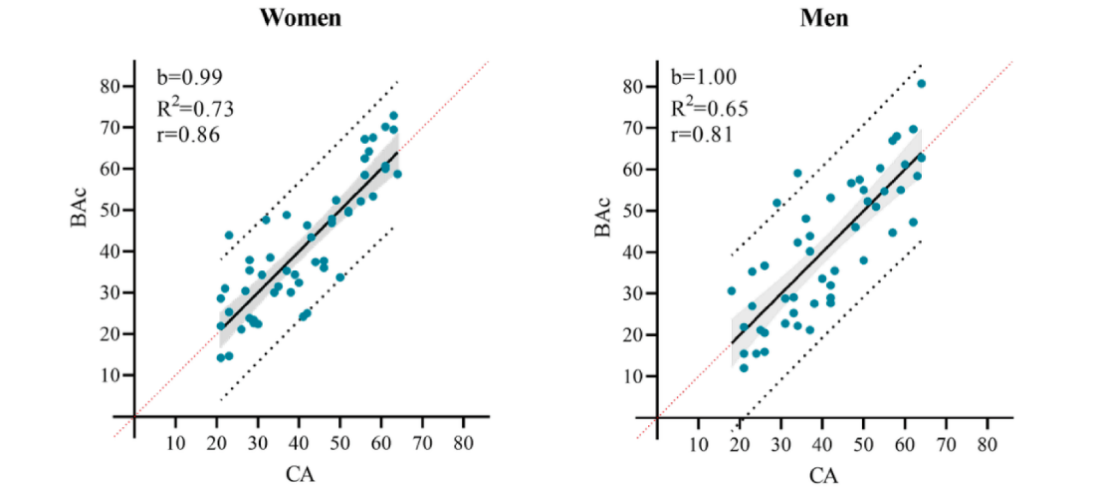
The BAc regression lines for women and men, respectively with 95% Confidence interval (shaded area), 95% Prediction intervals (black dotted lines) and line of identity (red dotted line). Slope (b), correlation coefficient (r) and coefficient of determination (R2).

**Figure 5:**
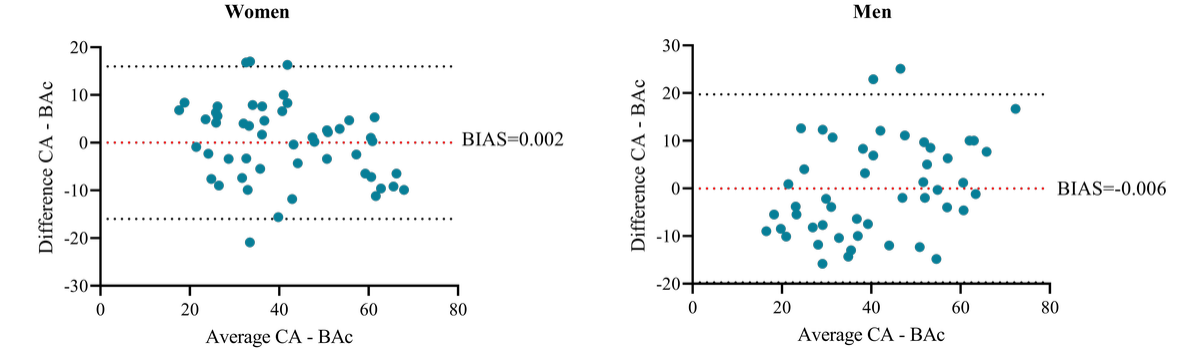
Bland Altman plot for women and men, respectively with BIAS (red dotted line), upper and lower limits of agreement (black dotted lines).

The corrections are visualized in Figure 3, showing how the overestimation of BA in younger adults and underestimation of older adults are attenuated. In addition, Figure 4 visualizes the regression of BAc on CA (R^2^=0.73; *P*<.001 and R^2^=0.65; *P*<.001). BAc is scattered relatively close and symmetrically above and below the regression line with a standard error of the estimate of 8.2 years (women) and 10.2 years (men). We found no statistical difference between mean CA and mean BAc in women (*P*=.99) or men (*P*=.99). To assess the agreement between CA and BAc, we made a Bland-Altman plot and found a mean difference of 0.002 in women and – 0.006 in men, respectively (Figure 5).

## Discussion

### Principal Findings

In this study, we aimed to develop a BA model, able to measure healthy aging trajectory, using simple, clinically relevant biomarkers that would respond to changes in health behavior. We selected 9 biomarkers listed in Table 3 and applied PCA to estimate individual BA. The 9 biomarkers represent metabolic health (HDL, TC, and adiponectin) and bodily functions (FEV_1_, MAP, and suPAR), and include very important clinical age–related variables (VO_2max_, HbA_1c_, and waist circumference) [28]. We found no difference between BAc and CA in the healthy reference group of women and men, and the BA model for both women and men showed a high linear relationship with CA. The disagreement between CA and BAc was low and unbiased. A higher variation in the BA model for men resulted in a lower coefficient of determination (R^2^=0.65; *P*<.001) compared with the BA model for women (R^2^=0.73; *P*<.001).

Sex differences were also observed in the relative contribution of each biomarker to the BA estimate. This indicates that some biomarkers of aging are influenced by sexual dimorphism [47]. HDL, for example, contributes 15.2% ([0.39^2^/0.999] × 100) in women and a negligible 0.04% in men ([–0.02^2^/1.001] × 100). HDL levels are higher in women than in men of the same age [48]. However, during menopause HDL levels decrease (and LDL increase), thereby increasing the cumulative risk of CVD [49]. In general, the multifaceted effects of menopause on metabolism may imply that further development of the model should evaluate if separate models for pre- and postmenopausal women are required. Waist circumference contributed the most (24.1%) in the estimation of BA for men but only 3.0% in the estimation of BA for women. This agrees well with the sex difference in fat distribution—men have a relatively more central distribution of fat with aging even in the absence of weight gain [50]. By contrast, a similar deterioration of VO_2max_ and FEV_1_ between sexes is expected [47]. This was not the case in our study, as VO_2max_ and FEV_1_ contributed more to the BA model for men. This difference may be balanced by normalizing VO_2max_ and FEV_1_ to lean mass and height, respectively. In addition, the small sample size should be mentioned as a limitation in these observations.

The BA model is based on a healthy reference adult subsample of the population. However, in 8% (4/51) of the women and 16% (n=8/49) of the men, the age difference (CA – BAc) was more than +10 years (Figure 5). One of these women and 7 of these men stand out by having a BMI between 25 and 36 kg/m^2^. Because BMI is causally related to morbidity and mortality [51], it could be argued that individuals with a BMI over 24.9 kg/m^2^ are not suitable to be included in this study representing a healthy aging reference group. However, cardiorespiratory fitness (VO_2max_) may be an even better predictor for CVD and premature all-cause mortality [52]. Further, a better VO_2max_ was found to attenuate the risks related to overweight and obesity [53,54]. The majority (41/51, 80%, and 46/49, 94%, of women and men, respectively) of the study participants adhered to the recommendations of a minimum of 150 minutes/week of moderate to vigorous physical activity and had a moderate to high cardiorespiratory fitness level [28]. Therefore, we did not use high BMI as exclusion criteria. Within this consideration, there also lies an effort to recruit a subsample of the population representing normal healthy aging instead of an extremely healthy and active subsample often more prone to participate.

### Comparison With Previous Work

In our data set, the highest correlated biomarker with CA was MAP (*r*=0.51; *P*<.001). MAP reflects vascular resistance and BP measurements are the commonly used biomarkers in BA studies [1,4,32,37,55]. However, in contrast to our study, pulmonary function (FEV_1_ and FVC) consistently appears as the most significant parameter related to CA in these former studies [1,4,32,37,55]. In our study, FEV_1_ only appears as the third most correlated biomarker (*r*=–0.38; *P*<.001). A possible explanation is that the biomarkers used for BA estimations rely on register-based data collected in the mid- and late 20th century, primarily representing individuals from Asia and the United States. Thus, it reflects a certain time era and population behavior, for example, regarding smoking prevalence, which has decreased since then [56]. Finally, it is important also to take into account the difference in health behavior seen between ethnic groups.

To estimate BA, we used the 1PC as a general aging factor. In the field of BA prediction models, PCA is considered an improvement compared with multiple linear regression [31]. Even so, PCA is still a linear model, thereby assuming that biomarkers change linearly throughout the age span [57]. While many biomarkers are assumed to decline with a slope of 1% per year [58], some biomarkers may deviate from this linearity, especially toward the higher end of the age span. The proportions of total variance explained by the PCA in our study (31% and 25% women and men, respectively) were similar to those found in other studies using the 1PC, varying from 23% to 42% [3,12,30,32] in women and from 20% to 37% in men [3,12,30,31,37,55]. These studies found that using PCA was valid and clinically useful. However, recent studies [5,34,36] comparing different models found that the Klemera and Doubal model (KDM) [59] was superior at predicting mortality outcomes [60]. Keeping in mind that these results also depend on the specific set of biomarkers included, the algorithm from the KDM should be included in future research on the present BA estimation.

### Future Research

This is a first-generation model which is why this work should be used to initiate further research to understand the interpretation of the model fully. Larger sample size is necessary to do a proper sensitivity analysis on how changes in each biomarker affect the BA estimate. In addition, a larger sample size would improve the validity of the selected biomarkers. In this study, the biomarkers were selected based on their significant correlation with CA in a cross-sectional analysis. Using cross-sectional data provides information on the age difference in the biomarkers at a specific point in time. To improve the statistical validity of the measures selected as biomarkers, a significant longitudinal correlation with CA should be investigated. This way the age difference in the biomarkers can be assessed over time [9].

Applying the BA model to longitudinal data is an important future investigation, to see if a relatively high BA is a predictor of poor health outcomes such as T2D, CVD, and mortality. Furthermore, investigating the BA model in health-related interventions will provide evidence as to whether the model can be used as a valid clinical tool for measuring disease risks. Our study has strength in its reproducibility—a key element for BA applicability. The majority of the 9 biomarkers are common measurements in the clinic and in science, where standard quantitative techniques are used. Thus, quantifying BA by the combination of these 9 biomarkers has the advantage of being less susceptible to artifactual variations related to the method of measurement and being accessible from stored plasma samples and databases in national health registers. That being said, the feasibility of measuring suPAR and adiponectin in regular clinical routine is low. Thus, future studies should investigate how the exclusion of suPAR and adiponectin affects the ability of the BA model to identify high-risk individuals and to assess the effect of health-enhancing interventions.

### Conclusions

The 9 physiological variables identified in this study as aging biomarkers are highly relevant to assess age-related changes affecting the risk of disease and physical capacity. The BA model has potential for clinical use, due to low technical difficulty and minimally invasive techniques. Estimation of BA has potential as an outcome measure in health-promoting interventions and as a pedagogical aid. Future research is required to investigate how the model will work in populations deviating from the healthy aging spectrum (eg, in individuals with T2D, CVD, or low cardiorespiratory fitness). We expect that the indicator of being biologically old is easy to understand, as a risk of disease and premature mortality, which explains why this indicator might drive individual motivation toward a healthier lifestyle. However, work remains to be performed to improve the model’s validity as a clinical tool and its predictive abilities including, but not restricted to, its reanalysis in a much larger sample size, test-retest reliability, and assessment of the longitudinal stability of the biomarkers.

## Appendix

Multimedia Appendix 1 Correlation coefficient with chronological age for the nine measurements included as biomarkers in the BA model. (A) Waist circumference (cm), (B) High Density Lipoprotein (HDL) (mmol/L), (C) Forced Expiratory Volume in the first second (FEV_1_) (L), (D) Maximal oxygen consumption (VO_2max_) (ml/min/kg), (E) Total cholesterol concentration (mmol/L), (F) Mean Arterial Pressure (MAP) (mmHg), (G) Glycated hemoglobin (HbA_1c_) (mmol/mol), (H) Adiponectin (mg/ml), (I) soluble urokinase plasminogen activator receptor (suPAR) (ng/ml).

Multimedia Appendix 2 Candidate biomarkers measured in the study participants (n=100) and their correlation with age.

## Abbreviations

1PC: first principal component
BA: biological age
BAc: corrected biological age
BAS: biological age score
BP: blood pressure
CA: chronological age
DBP: diastolic blood pressure
FBG: fasting blood glucose
FEV_1_: forced expiratory volume in 1 second
FVC: forced vital capacity
HbA_1c_: glycated hemoglobin
HDL: high-density lipoprotein
KDM: Klemera and Doubal model
LDL: low-density lipoprotein.
MAP: mean arterial pressure
PCA: principal component analysis
SBP: systolic blood pressure
SF-12: 12-item Short Form
suPAR: soluble urokinase plasminogen activator receptor
T2D: type 2 diabetes mellitus
TC: total cholesterol
VO_2max_: maximal oxygen consumption
