# Correction: A Model for Estimating Biological Age From Physiological Biomarkers of Healthy Aging: Cross-sectional Study

**DOI:** 10.2196/40508

**Published:** 2022-06-28

**Authors:** Karina Louise Skov Husted, Andreas Brink-Kjær, Mathilde Fogelstrøm, Pernille Hulst, Akita Bleibach, Kaj-Åge Henneberg, Helge Bjarup Dissing Sørensen, Flemming Dela, Jens Christian Brings Jacobsen, Jørn Wulff Helge

**Affiliations:** 1 Xlab, Center for Healthy Aging Department of Biomedical Sciences University of Copenhagen Copenhagen Denmark; 2 Department of Physiotherapy and Occupational Therapy University College Copenhagen Copenhagen Denmark; 3 Digital Health Department of Health Technology Technical University of Denmark Lyngby Denmark; 4 Biomedical Engineering Department of Health Technology Technical University of Denmark Lyngby Denmark; 5 Department of Geriatrics Bispebjerg and Frederiksberg Hospital Copenhagen Denmark; 6 Department of Biomedical Sciences Faculty of Health and Medical Sciences University of Copenhagen Copenhagen Denmark

In “A Model for Estimating Biological Age From Physiological Biomarkers of Healthy Aging: Cross-sectional Study” (JMIR Aging 2022;5(2):e35696) the authors noted one error.

In the originally published article, Figure 4 inadvertently appeared with the same image as that of Figure 3. In the corrected version of the article, Figure 4 was updated with the following image:

**Figure 4:**
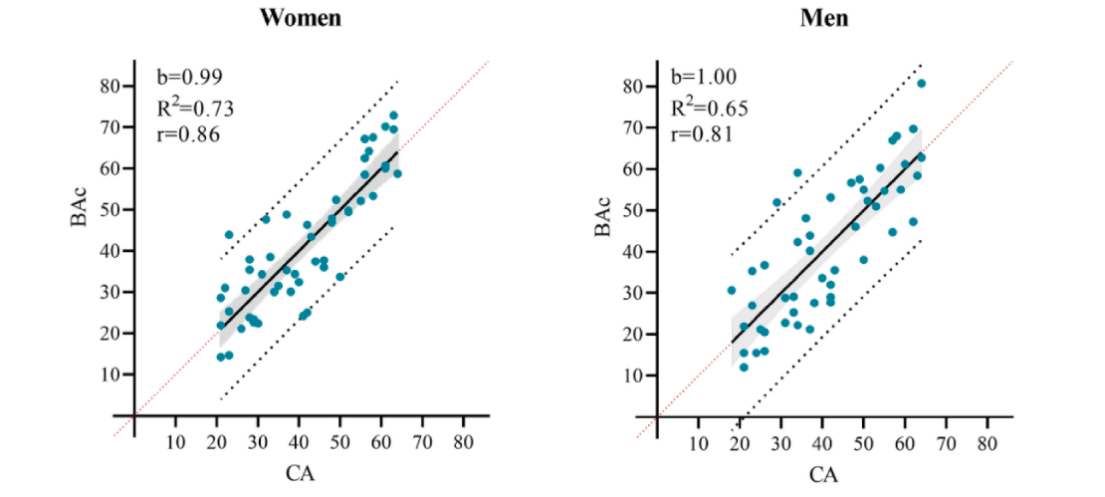
The BAc regression lines for women and men, respectively with 95% Confidence interval (shaded area), 95% Prediction intervals (black dotted lines) and line of identity (red dotted line). Slope (b), correlation coefficient (r) and coefficient of determination (R2).

The correction will appear in the online version of the paper on the JMIR Publications website on June 28, 2022, together with the publication of this correction notice. Because this was made after submission to PubMed, PubMed Central, and other full-text repositories, the corrected article has also been resubmitted to those repositories.

