# Supplementary figures and images for: A Model for Estimating Biological Age From Physiological Biomarkers of Healthy Aging: Cross-sectional Study
Source: JMIR Aging. 2022 May 10;5(2):e35696. doi: 10.2196/35696 (PMC9131142; doi:10.2196/35696)

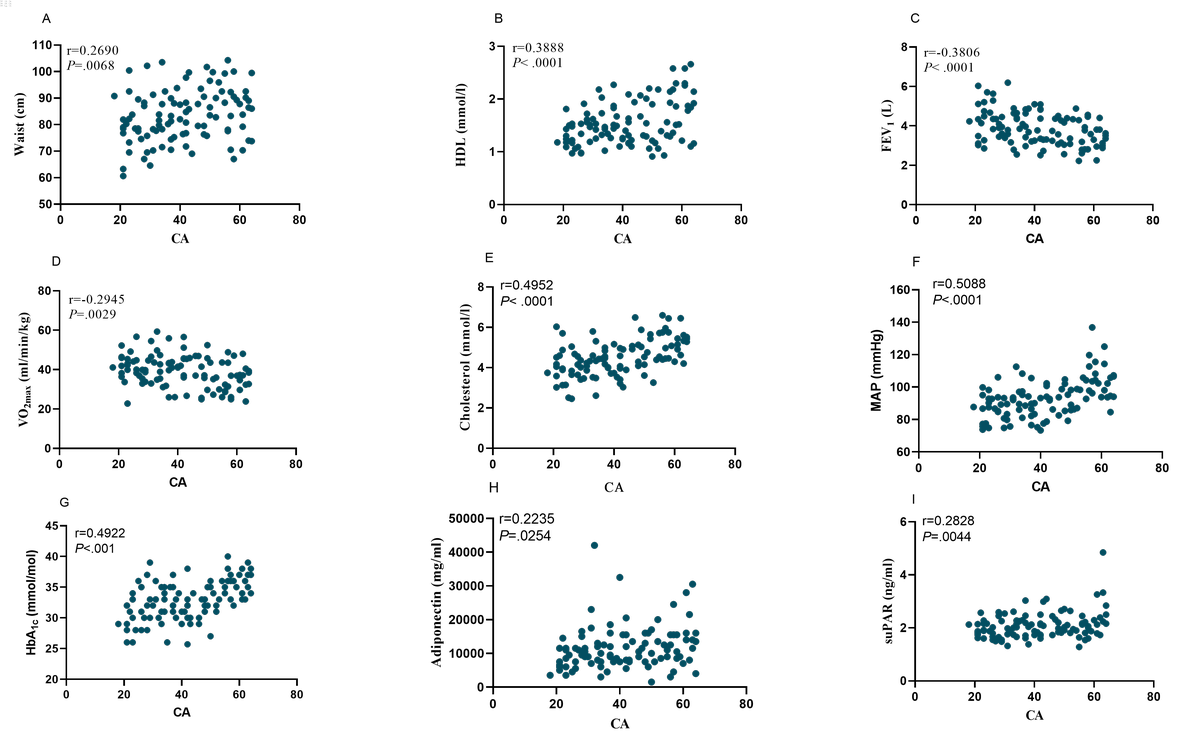

Supplement: Multimedia Appendix 1 [file aging_v5i2e35696_app1.png]
