# Supplementary material for: A Model for Estimating Biological Age From Physiological Biomarkers of Healthy Aging: Cross-sectional Study
Source: JMIR Aging. 2022 May 10;5(2):e35696. doi: 10.2196/35696 (PMC9131142; doi:10.2196/35696)
Supplement: Multimedia Appendix 2 [file aging_v5i2e35696_app2.docx]

| Candidate biomarkers measured in the study participants (n=100) and their correlation with age. | | |
| --- | --- | --- |
| Biomarkers | Pearson's r | *P* |
|  |  |  |
| **Body composition** |  |  |
|  |  |  |
| (1) Weight, *kg* | 0.028 | .79 |
| (2) Waist circumference, *cm* | 0.269 | .007 |
| (3) Hip circumference, *cm* | -0.003 | .98 |
| (4) Waist/Hip ratio | 0.365 | .0002 |
| (5) Fat mass, *%* | 0.156 | .12 |
| (6) Muscle mass, *kg* | -0.068 | .50 |
|  |  |  |
| **Metabolic health** |  |  |
|  |  |  |
| (7) Fasting blood glucose, *mmol/l* | 0.315 | .001 |
| (8) HbA1c, *mmol/mol* | 0.492 | <.0001 |
| (9) Insulin, *pmol/l* | 0.026 | .79 |
| (10) Triglycerides, *mmol/l* | 0.077 | .44 |
| (11) Free fatty acids, *μmol/l* | 0.152 | .13 |
| (12) Leptin, pn/mL | -0.086 | .40 |
| (13) Adiponectin, *mg/ml* | 0.224 | .03 |
| (14) HDL, *mmol/l* | 0.389 | <.0001 |
| (15) LDL, *mmol/l* | 0.370 | .0002 |
| (16) CHOL, *mmol/l* | 0.495 | <.0001 |
| (17) CHOL/HDL ratio | 0.044 | .66 |
|  |  |  |
| **Immune function** |  |  |
|  |  |  |
| (18) CRP, *mg/l* | -0.169 | 0.12 |
| (19) suPAR *ng/ml* | 0.283 | .004 |
|  |  |  |
| **Cell blood count** |  |  |
|  |  |  |
| (20) Hemoglobin, *mmol/l* | 0.070 | 0.49 |
| (21) Hematocrit, *%* | 0.112 | 0.28 |
|  |  |  |
| **Cardiorespiratory function** |  |  |
|  |  |  |
| (22) Diastolic BP | 0.512 | <.0001 |
| (23) Systolic BP | 0.451 | <.0001 |
| (24) FEV1, *L* | -0.381 | <.0001 |
| (25) FVC, *L* | -0.316 | 0.001 |
| (26) FEV1/FVC, *%* | -0.305 | 0.002 |
|  |  |  |
| **Physical capacity** |  |  |
|  |  |  |
| (27) VO_2_max, *ml/min/kg* | -0.294 | 0.003 |
| (28) STS, *stands* | -0.176 | 0.08 |
| (29) Handgrip strength, *kg* | -0.115 | 0.25 |
| (30) Bicep strength, *kg* | -0.155 | 0.13 |
| (31) Quadriceps strength, *Nm* | -0.174 | 0.03 |
|  |  |  |
| HbA1c; g*lycosylated hemoglobin type A1c,* AGEs*; Advanced glycation end products,* HDL; *High density lipoprotein*, LDL; *Low density lipoprotein*, CHOL; *Total cholesterol*, CRP; *C-reactive protein,* suPAR; *soluble urokinase plasminogen activator receptor*, BP; *Blood pressure*, FEV1; *Forced expiratory volume in first second*, FVC; *Forced vital capacity*, STS; *30 sec.* *sit-to-stand chair rise*, VO_2_max; *maximal oxygen consumption. Missing values was present in CRP (n=87), Hematocrit (n=97) and Hemoglobin (n=99) and Bicep strength (n=98).* | | |
